# Accuracy and Precision Evaluation of International Standard Spherical Model by Digital Dental Scanners

**DOI:** 10.1155/2020/1714642

**Published:** 2020-12-09

**Authors:** Hong Xin Cai, Qi Jia, HaoYu Shi, Yujie Jiang, Jingnan Xue, ChunXu Chen, Haotian Gong, Jie Liu, Eui-Seok Lee, Heng Bo Jiang

**Affiliations:** ^1^Stomatological Materials Laboratory, School of Stomatology, Shandong First Medical University & Shandong Academy of Medical Sciences, Tai'an, Shandong 271016, China; ^2^Department of Oral and Maxillofacial Surgery, Graduate School of Clinical Dentistry, Korea University, Seoul 08308, Republic of Korea

## Abstract

With the popularization of digital technology and the exposure of traditional technology's defects, computer-aided design and computer-aided manufacturing (CAD/CAM) has been widely used in the field of dentistry. And the accuracy of the scanning system determines the ultimate accuracy of the prosthesis, which is a very important part of CAD/CAM, so we decided to evaluate the accuracy of the intraoral and extraoral scanners. In this study, we selected the sphere model as the scanning object and obtained the final result through data analysis and 3D fitting. In terms of trueness and precision, the scanner of SHINING was significantly different from that of others; however, there was no significant difference between TRIOS and CEREC. SHINING showed the lowest level of accuracy, with CEREC slightly lower than TRIOS. The sphere model has also been proven to be scanned successfully.

## 1. Introduction

With the emergence of digitalization, CAD/CAM systems [1] have found an increasingly wide utilization in the field of prosthodontics on account of its considerable strengths [2]. Given the fact that the traditional prostheses gradually fail to meet the needs of patients, the use of intraoral [3] and extraoral scanners in dentistry is becoming more and more common [4, 5].

By using the specific and intuitive model presented from intraoral and extraoral scanners [6–9], we can immediately obtain detailed information of the patient's oral cavity and its digital files. And the files will be imported into the computer to complete the design and production [10]. The extraoral scanner scans the impression model, and the intraoral scanner directly scans the patient's oral cavity [7]. Compared to the traditional technology, the scanning technology is undoubtedly time-saving and efficient [11, 12].

There are many factors that affect the scanning results and data collection. Recent studies have shown that different scanning strategies can affect the accuracy of results [13]. It has been reported that different scanning systems also contribute to different experimental results [14]. In addition, the selection of the impression materials and operation time will affect the accuracy of extraoral scanners, while the scanning range, light [15], and oral tissue will affect intraoral scanners [16, 17].

Considering the above factors, it is of clinical significance to evaluate the accuracy of intraoral and extraoral scanners [9]. The early Flügge's study claimed that the extraoral scanners performed better than intraoral scanners under the oral environment [13]. However, as the algorithms and scanning techniques evolve, Tomita et al. concluded that the intraoral scanners had higher accuracy than extraoral scanners as they were studying their self-manufactured denture model *in vitro* [18]. Further, the performance of the intraoral scanner is significantly influenced by the geometry of the scanned object, for the fact that it accumulates the scanned image and records the scanning path of the object to obtain the complete image. Especially, the uniformity of the model will also reduce the performance of the intraoral scanner. Therefore, this study is based on an international standard model to evaluate the accuracy of intraoral and extraoral scanners [19, 20].

After looking up a lot of literature, we found that there were few papers based on the sphere model [19], so this experiment adopted the ISO standard sphere model [21]. According to the definition of accuracy regulated in ISO 5725 including trueness and precision, the trueness refers to the degree of uniformity between a measurement result and the reference value, while the latter one maintains the uniformity between independent measurement results. According to the standard in ISO 3290-2, we chose a sphere with Grade 20 to conduct the experiment. And the error of diameter and surface less than 0.001 mm can be ignored in this grade [22].

## 2. Materials and Methods

### 2.1. Fabrication of Models

**Figure 1:**
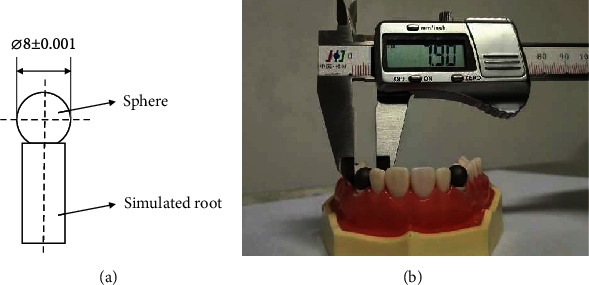
Design drawings of the (a) sphere model, and (b) optical image.

According to the clinical practice, the theoretical value of the diameter of the sphere model based on the ISO 12836 [21] was set as 8 mm, as shown in Figure 1(a). The 3D file of the model was drawn by computer-aided design software (AutoCAD 2018, Autodesk, USA), which was exported in stereolithography (STL) format. The test model was made of stainless steel, and we used computer numerical controlled (CNC) milling to fabricate it. Moreover, the model was sandblasted with a powder size of 80 *μ*m as specified by the international standard.

### 2.2. Creation of Gold Standard Values

Thirty measurements of the sphere model's diameter were taken by Vernier Calipers, the average of which was taken as the truth value in this paper. One of the measurements was shown in Figure 1(b). The true value was set as the gold standard for evaluation, and the 3D file based on the value was created as the gold standard file for 3D fitting.

### 2.3. Scanning Process

One extraoral scanner (SHINING D200+, CHN) and two intraoral scanners (CEREC AC D3492, Sirona, GER and TRIOS T12A, 3Shape, DEN), respectively, performed 30 scans of the sphere model and saved them as STL files. The model was scanned by the same skilled technician who followed the scanning method recommended by the different instrument manufacturers to eliminate interference and improve feasibility [7].

### 2.4. Data Acquisition and Image Matching

The scanned original files were converted into STL files, which were then imported into a reverse engineering software (Geomagic Control X 2018, 3D SYSTEMS, USA). The test indicator (diameter) was measured and recorded through the software, and the 3D files of test groups were compared with the gold standard files for 3D fitting. Meanwhile, root mean square error (RMSE) values were recorded.

### 2.5. Mathematical Analysis

The formula for accuracy assessment is as follows [23]:

(1) $$\begin{array}{c} \text{Trueness}=\left|\left(d_{R}-d_{M}\right)\right|, \end{array}$$

(2) $$\begin{array}{c} \text{Precision}=\left|\left(d_{A}-d_{M}\right)\right|, \end{array}$$

(3) $$\begin{array}{c} \Delta d_{M}=\left|\left(d_{R}-d_{M}\right)/d_{R}\right|, \end{array}$$

(4) $$\begin{array}{c} \Delta S\left(d_{M}\right)=\left|S\left(d_{M}\right)/d_{R}\right|, \end{array}$$

Perform statistical analysis on scanned data using SPSS v.20.0 (IBM, USA). The data conform to a normal distribution, but they are not conformable in the homogeneity of the variance. The Nonparametric Kruskal-Wallis test analyzed the difference in parameters and the result of *p* < 0.01 attested statistically significant differences.

## 3. Results

### 3.1. Absolute Mean Trueness and Precision of the Sphere Model

**Figure 2:**
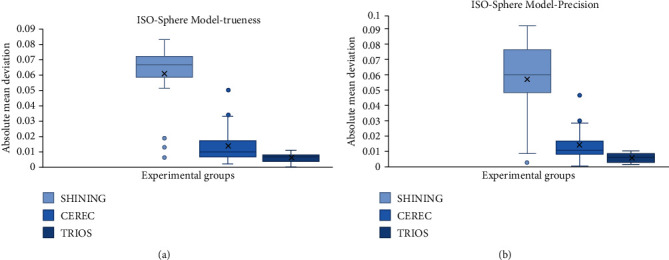
Boxplots of absolute mean trueness (a) and precision values (b) of the sphere model. The horizontal line of each Boxplot from top to bottom represents the upper edge, upper quartile, median, lower quartile, and lower edge of the value, respectively, in addition the round dots represent outliers, and the crosses represent averages.

The value of absolute mean trueness and precision of the sphere model is enumerated in Table 1.  Figure 2(a) shows boxplots of the absolute mean trueness values, and Figure 2(b) shows boxplots of the absolute mean precision values of the sphere model. Regarding diameter errors of the sphere model, the TRIOS reveals the highest trueness and precision, respectively. The comparison between the intraoral scanners and the extraoral scanners shows that compared to the CEREC and SHINING, the mean trueness value of TRIOS is much lower. The mean precision value of TRIOS is significantly lower than CEREC and SHINING.

### 3.2. Relative Errors of Sphere Model

Table 2 shows the relative errors of the trueness and precision of the sphere model. The relative errors of the sphere model are less than 0.008 mm, respectively. This is an ideal result that when comparing the relative errors of trueness and precision between these scanners, the trend is similar to that of the absolute mean deviation of trueness and precision.

### 3.3. Broken Line Graph and RMSE

**Figure 3:**
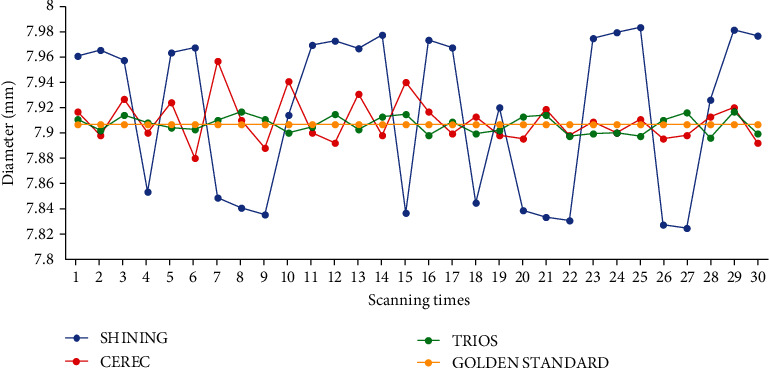
The broken line graph of the original data of the sphere model diameter measured by scanners. The fluctuation range and frequency of the tree broken lines are different and represent different meanings. (The yellow line used for comparison means the gold standard.)

The broken line graph of the original data of the sphere model diameter measured by scanners is shown in Figure 3. The yellow line is the gold standard. It is obvious that SHINING's values are not concentrated and far from the yellow line. We found that the values of CEREC and TRIOS were very close to the gold standard, also highly centralized and accurately combined with RMSE in Table 3.

### 3.4. 3D-Fitting Measurement

**Figure 4:**
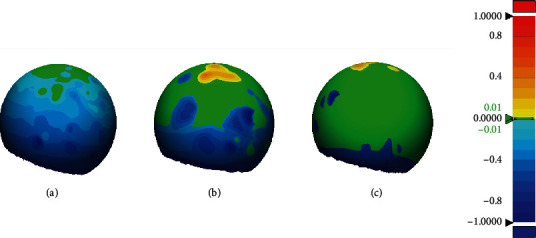
3D-fitting comparison of digital impression files ((a) SHINING, (b) CEREC, and (c) TRIOS) with gold standard file. Within the specified accuracy range, green represents the overlap between test groups and control group, yellow represents higher than the control group, and blue represents lower than the control group. The greener areas mean the more accurate the scanned image.

Figure 4 shows a 3D-fitting comparison of the digital impression between test groups and the control group. The difference in colors other than green represents the difference between the measured value and the gold standard. More green areas mean the scanned image more accurate. The results show that there is a larger error of SHINING compared with other scanners, and a large area is lower than the control group. The fitting result of TRIOS is the best with the largest green area.

### 3.5. Analysis of Significant Difference

We find that there is no significant difference between CEREC and TRIOS, but the absolute mean precision values of the CEREC are significantly lower than TRIOS through statistical analysis. And SHINING is different from CEREC and TRIOS in terms of precision and trueness obviously.

## 4. Discussion

In this study, the accuracy of intraoral and extraoral scanners was evaluated by a sphere model described in ISO 12836 [21]. The sphere model is of positive geometry with a precision limited to 0.0002 mm, made of dimensionally stable stainless steel. There have been many studies evaluating the accuracy of scanners by Inlay-cavity die and Grown-and-bridge die, but few studies are based on the sphere model because it is difficult to be identified by the intraoral scanner.

Arakida et al. assessed the influence of ambient light illumination and color temperature on the authenticity and accuracy of a digital signal and proved that the conditions of 3900 k and 500 lux are the most suitable conditions for digital scanning [24]. Sun et al. compared the reproducibility of intraoral scanners *in vivo* and *in vitro*, respectively, and the results showed that the reproducibility was comparable, so different scanning environment will affect the scanning results of intraoral scanners [25].

According to the scanning standard described in ISO 12836 [21], the scanning process of each scanner was executed 30 times with room temperature of (23 ± 2)°C and the change of temperature controlled within ±1°C, which we had followed to minimize the error caused by external interference.

There are still many factors like equipment or manual operations that can affect the accuracy of the scanner [26]. Therefore, we designed some schemes aimed at these problems to minimize their impact on accuracy. The scanning principle and operation methods of intraoral and extraoral scanners in this experiment are different. And we added a base at the bottom of the sphere model for proper operation. If the operator had touched the sphere directly by hand in the experiment, the surface of the model would have changed greatly and thus the final result would have been affected. Thus, it is necessary to add the base which can also fix the sphere model to make scanning easier.

Reproducibility and repeatability are also important components of the evaluation [27]. Reproducibility can be defined as the consistency of the measured results of the same object under different conditions, while repeatability is affected by operating, timing, equipment, etc. Therefore, this experiment was carried out by a skilled operator in strict accordance with the methods specified by the scanner manufacturers to reduce the influence of operation.

There is no significant difference between values obtained in the experiment and the standard values created from the model, so it is feasible to scan the sphere model with these three scanners.

There are limitations to our experiments as well. The long operation of the scanner may result in the degradation of the performance, which may affect the scanning results of the latter part. And the choice of a single model has some limitations in the evaluation of scanners. In terms of the clinic, due to the complex oral environment, like saliva, blood, and soft tissue, there will be deviations in the actual operation [28, 29]. Additionally, the level of analysis and measurement software used also affects the results.

According to the study of Burzynski et al., with the progress of intraoral scanning technology, the shrinking of the camera, and the acceleration of acquisition time, patients may show a greater preference for digital impression [30]. Through investigation, Ahmed et al. found that with the development of digitization and the improvement of scanner accuracy, people's acceptance of using CAD/CAM for oral disease treatment has gradually increased [31]. Alghazzawi argued that the coming trend for most practitioners would be the use of an acquisition camera attached to a computer that was equipped with appropriate software and the capability of forwarding the image to the laboratory [32].

Our experiment compared one extraoral scanner (SHINING) with two intraoral scanners (CEREC, TRIOS), whose results showed that the accuracy of CEREC was between the other two. It indicated that the intraoral scanners were better than the extraoral one. Since no controversy was shown in the results of this experiment, the results of clinical trials of the extraoral and intraoral scanners need further discussion.

## 5. Conclusions

The accuracy of the intraoral scanners in this experiment was greater than that of the extraoral scanner. The intraoral scanner has certain reliability in practical application and is worth to trial in clinics. The sphere model is achievable as a scanning object, but it requires further research and improvement.

## Figures and Tables

**Table 1:** Mean trueness/precision values ± standard deviation (SD).

Test group	Trueness (mm)	Precision (mm)
SHINING	0.061 ± 0.018	0.059 ± 0.023
CEREC	0.010 ± 0.010	0.014 ± 0.010
TRIOS	0.006 ± 0.003	0.006 ± 0.003
True value	7.908	

**Table 2:** Relative errors of the trueness and precision of the sphere model (diameter).

Test group	Trueness (mm)	Precision (mm)
SHINING	0.008	0.008
CEREC	0.002	0.002
TRIOS	0.001	0.001

**Table 3:** RMSE values for the 3D-fitting results.

Test group	RMSE
SHINING	0.064
CEREC	0.017
TRIOS	0.007

## Data Availability

The data used to support the findings of this study are included within the article.
